# Comparative Transcriptome Profile of the Cytoplasmic Male Sterile and Fertile Floral Buds of Radish (*Raphanus sativus* L.)

**DOI:** 10.3390/ijms17010042

**Published:** 2016-01-06

**Authors:** Shiyong Mei, Touming Liu, Zhiwei Wang

**Affiliations:** 1 College of Life Sciences, Wuhan University, Wuhan 430072, China; 2 Institute of Bast Fiber Crops and Center of Southern Economic Crops, Chinese Academy of Agricultural Sciences, Changsha 410205, China; liutouming@caas.cn; 3 CAS Key Laboratory of Plant Germplasm Enhancement and Specialty Agriculture, Wuhan Botanical Garden, Chinese Academy of Sciences, Wuhan 430074, China; wangzhiwei@wbgcas.cn

**Keywords:** cytoplasmic male sterility, radish, Illumina sequencing, transcriptome, differentially expressed genes

## Abstract

Radish cytoplasmic male sterility (CMS) has been widely used for breeding in *Raphanus* and *Brassica* genera. However, the detailed regulation network of the male sterility remains to be determined. Our previous work has shown that the abnormalities in a CMS radish appeared shortly after the tetrad stage when microspores were malformed and the tapetal cells grew abnormally large. In this work, histological analysis shows that anthers are at the tetrad stage when the radish buds are about 1.5 mm in length. Furthermore, a high throughput RNA sequencing technology was employed to characterize the transcriptome of radish buds with length about 1.5 mm from two CMS lines possessing the CMS-inducing *orf138* gene and corresponding near-isogenic maintainer lines. A total of 67,140 unigenes were functionally annotated. Functional terms for these genes are significantly enriched in 55 Gene Ontology (GO) groups and 323 Kyoto Encyclopedia of Genes and Genomes (KEGG) pathways. The transcriptome detected transcripts for 72 out of a total of 79 protein genes encoded in the chloroplast genome from radish. In contrast, the radish mitochondrial genome contains 34 protein genes, but only 16 protein transcripts were detected from the transcriptome. The transcriptome comparison between CMS and near-isogenic maintainer lines revealed 539 differentially expressed genes (DEGs), indicating that the false positive rate for comparative transcriptome profiling was clearly decreased using two groups of CMS/maintainer lines with different nuclear background. The level of 127 transcripts was increased and 412 transcripts were decreased in the CMS lines. No change in levels of transcripts except CMS-inducing *orf138* was identified from the mitochondrial and chloroplast genomes. Some DEGs which would be associated with the CMS, encoding MYB and bHLH transcription factors, pentatricopeptide repeat (PPR) proteins, heat shock transcription factors (HSFs) and heat shock proteins (HSPs), are discussed. The transcriptome dataset and comparative analysis will provide an important resource for further understanding anther development, the CMS mechanism and to improve molecular breeding in radish.

## 1. Introduction

Plant cytoplasmic male sterility (CMS) is a maternally inherited trait preventing the production of functional pollen but maintaining female fertility. CMS has been observed in many crops and widely applied to produce hybrids with significant heterosis [1]. CMS is usually associated with chimeric open reading frames caused by mitochondrial genome reorganization [2,3,4]. In many cases, pollen fertility of the CMS plants can be restored by corresponding nuclear-encoded fertility restorer genes suggesting that CMS is an ideal model system to study nuclear-cytoplasmic gene interaction [5].

Considerable progress has been made in the molecular dissection of CMS and fertility restoration in plants. Many novel chimeric mitochondrial genes have been identified and are responsible for CMS through a variety of mechanisms (such as *urf13* from maize, *pcf* from petunia, *orf79/orfH79* from rice, *orf224/orf222* from rapeseed *etc.*) [6,7,8,9,10,11]. Recently, a new mitochondrial gene, *WA352*, was found to confer CMS in rice. WA352 interacts with the nuclear-encoded mitochondrial protein OsCOX11 which has a role in hydrogen peroxide degradation. The interaction between WA352 and OsCOX11 triggers an early reactive oxygen species (ROS) burst which promotes cytochrome c release to the cytosol, causing premature tapetal programmed cell death (PCD) [3]. Several fertility restorer (*Rf*) genes were isolated through map-based cloning strategy. The first cloned *Rf* gene encodes an aldehyde dehydrogenase [12]. *Rf17* and *Rf2* in rice encode acyl-carrier protein synthase-like protein and glycine-rich protein, respectively [13,14]. Sugar beet *Rf1* encodes the mitochondrial protein bvORF20 that resembles a yeast protease involved in mitochondrial protein quality control and interacts with the sterility-inducing factor preSATP6 [15,16]. All the other *Rf* genes encode pentatricopeptide repeat (PPR) proteins [8,17,18,19,20,21,22,23].

Radish (*Raphanus sativus* L.), an annual or biennial herb belonging to the family Brassicaceae, is an economically important root vegetable crop produced throughout the world. The edible taproot of radish is an excellent source of dietary fiber and carbohydrates, essential minerals and organic nutrients for humans [24,25,26]. Three different radish CMS germplasms, Ogura/Kosena, NWB and DCGMS CMSs, have been identified [18,20,27,28]. *Orf138/orf125* confers Ogura/Kosena CMS [18,20], and *orf463* is likely to be a causative factor for DCGMS CMS [28]. In recent years, next-generation sequencing technology obviously increased the efficiency and speed of gene discovery. By high-throughput cDNA sequencing (RNA-Seq), researchers can obtain almost the entire transcriptional landscape of gene expression in a high throughput and quantitative way. In this study, comparative transcriptome analysis of radish buds from CMS and their maintainer lines was conducted by Illumina sequencing, which might provide assistance for further deep analysis about anther development and CMS in radish.

## 2. Results and Discussion

### 2.1. The Floral Bud Size Is Associated with the Microspore Developmental Stage

**Figure 1:**
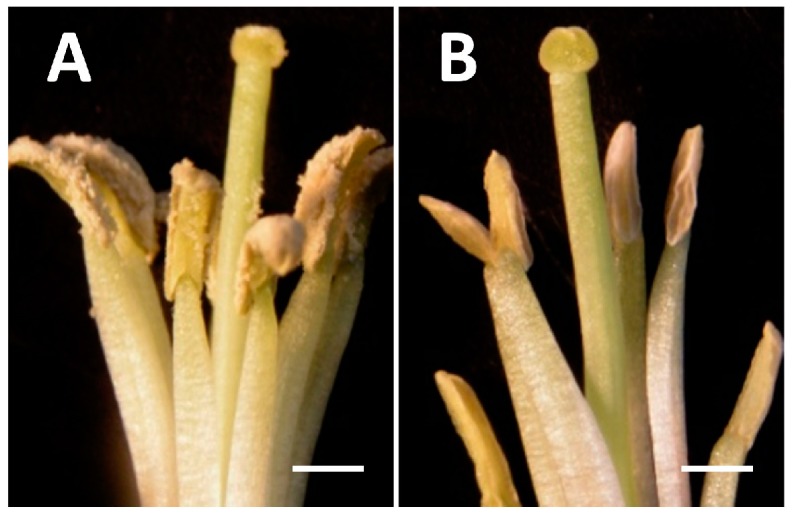
Partially dissected radish flowers. Male fertile plant (**A**); and male sterile plant (**B**). Bar = 1 mm.

**Figure 2:**
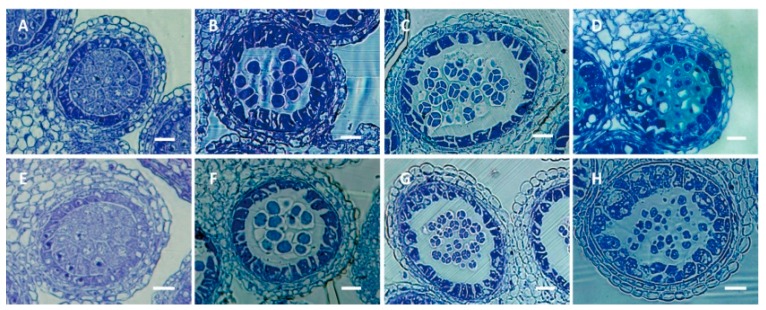
Development of anthers in the maintainer line HYBP-B (**A**–**D**) and the CMS line HYBP-A (**E**–**H**). Anther from floral bud at about 0.8 mm long (**A**,**E**); Anther from floral bud at about 1.1 mm long (**B**,**F**); Anther from floral bud at about 1.5 mm long (**C**,**G**); Anther from floral bud at about 2.0 mm long (**D**,**H**). Bar = 20 μm.

No morphological difference between the CMS line and the corresponding maintainer line was observed except for the anther. CMS lines have empty yellow anthers whereas maintainer lines have full and dehiscent anthers (Figure 1). No histological differences were observed between the CMS line 9802A1 and the corresponding maintainer line 9802B1 in the anther at the tetrad stage when tetrads were enclosed by a thick callose wall in our previous study [29]. After the tetrad stage, microspores released from tetrads underwent cell expansion and cell wall synthesis in male fertile anthers. In contrast, most microspores from CMS anthers grew slowly and were malformed, and the tapetal cell grew abnormally large with increased vacuoles [29]. The cytoplasm of 9802A1 was introgressed into HYBP-B and YH-B at more than 16 times of backcrossing. The resulting CMS lines, HYBP-A and YH-A, display different morphologies, indicating that they differ in their nuclear genomes. In this work, the stage of pollen abortion in HYBP-A and YH-A is the same with that of 9802A1 suggesting that different nuclear backgrounds could not change the pollen abortion phase from the same CMS cytoplasm. In the four lines, HYBP-A/B and YH-A/B, when the buds reached about 0.8 mm, anthers were at the microspore mother cell stage (Figure 2A). Microspore mother cells were undergoing meiosis when the buds were about 1.1 mm in length (Figure 2B). When the bud length was about 1.5 mm, individual tetrads could be seen (Figure 2C). In HYBP-B and YH-B, the phase of the vacuolate microspore appeared in buds about 2.0 mm in length (Figure 2D), with a single large vacuole displacing the single nucleus to one side of the cell.

### 2.2. Illumina Sequencing

Since distinct anther defects were histologically observed shortly after the tetrad stage in the CMS lines, we reckoned that the gene expression profiles at the tetrad stage could already change between the CMS lines and maintainer lines. The tetrad stage corresponded to floral buds about 1.5 mm in length from HYBP-A, HYBP-B, YH-A and YH-B. Thereafter, RNA of floral buds about 1.5 mm long was isolated from these four lines, and used for RNA-Seq. After trimming the raw data (93,800,000 reads), a total of 83,932,141 clean reads above the minimum quality threshold of Q20 per basewere selected with an average GC content of 47.7%.

### 2.3. De Novo Assembly and Functional Classification

High-quality clean reads were *de novo* assembled into 130,240 contigs with an average length of 570 bp and a N50 length of 821 bp. Sequences that did not extend on either end were defined as unigenes. In total, 67,140 unigenes showed similarity to known genes in at least one of the five public databases including NR, Swiss-Prot, GO, KOG (eukaryotic ortholog groups) and KEGG.

**Figure 3:**
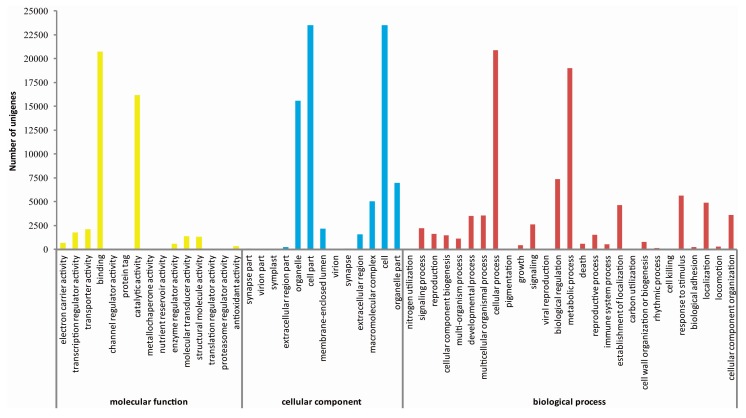
Gene ontology classifications of unigenes from the radish floral bud transcriptome. The *x*-axis indicates the sub-categories; the *y*-axis indicates the number of unigenes in a sub-category.

GO annotation including three ontologies (molecular function, cellular component and biological process) is an international gene classification system that provides a dynamically-updated standardized vocabulary for assigning functions of genes and their products [30]. BLAST2GO program was employed to find GO terms for assembled unigenes and 29,031 unigenes were assigned at least one GO term in 55 functional groups. Of these, most were assigned to the biological process ontology (86,785; 41.2%), followed by cellular component (78,749; 37.4%) and molecular function (45,090; 21.4%) ontologies (Figure 3).

**Figure 4:**
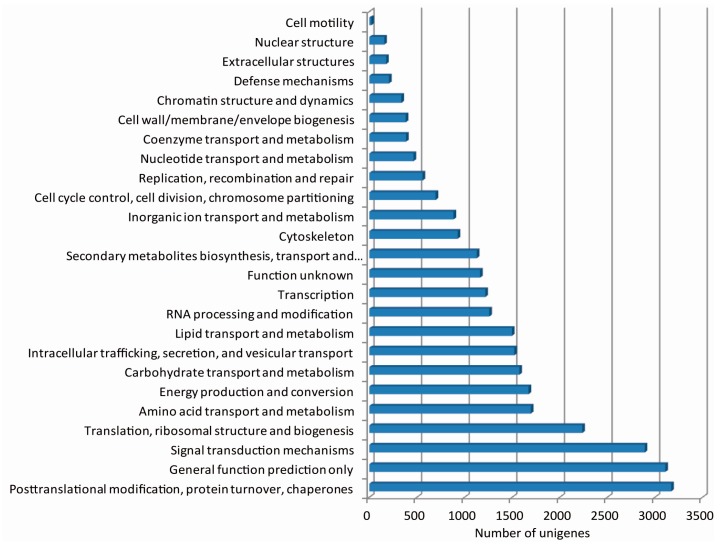
KOG function classification of unigenes from the radish floral bud transcriptome. The *x*-axis indicates the number of unigenes in a function class; the *y*-axis indicates the function classes.

The KOGs, based on orthologous relationships between genes, include proteins encoded by seven sequenced eukaryotic genomes [31]. In total, 26,527 unigenes were assigned to the KOG classification. Because some unigenes were annotated with multiple KOG functions, a total of 29,353 functional annotations were obtained. The four largest groups annotated were “posttranslational modification, protein turnover, chaperones” (3171 unigenes), “general function prediction only” (3110 unigenes), “signal transduction mechanisms” (2893 unigenes) and “Translation, ribosomal structure and biogenesis” (2237 unigenes) (Figure 4).

**Figure 5:**
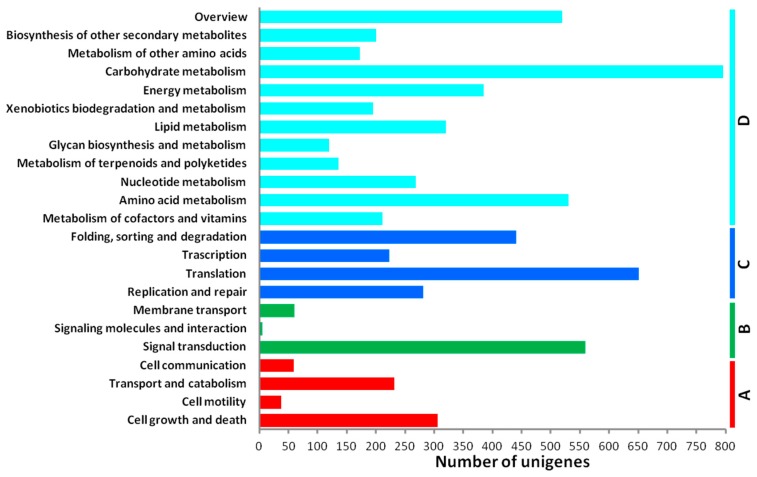
Classification based on categories of KEGG pathways. Cellular Processes (**A**); Environmental Information Processing (**B**); Genetic Information Processing (**C**); and Metabolism (**D**). The *x*-axis indicates the number of unigenes in a function class; the *y*-axis indicates the function classes.

The KEGG pathway records molecular interaction networks such as pathways and complexes. Sequence similarity search against KEGG GENES identified 5706 unigenes assigned to 323 KEGG pathways. The result showed that the three largest pathway categories were “carbohydrate metabolism” (803 members), “translation” (595 members) and “signal transduction” (563 members) suggesting that these pathways could be crucial for radish anther development (Figure 5).

### 2.4. Comparison of Gene Expression Levels between CMS and Maintainer Lines

**Figure 6:**
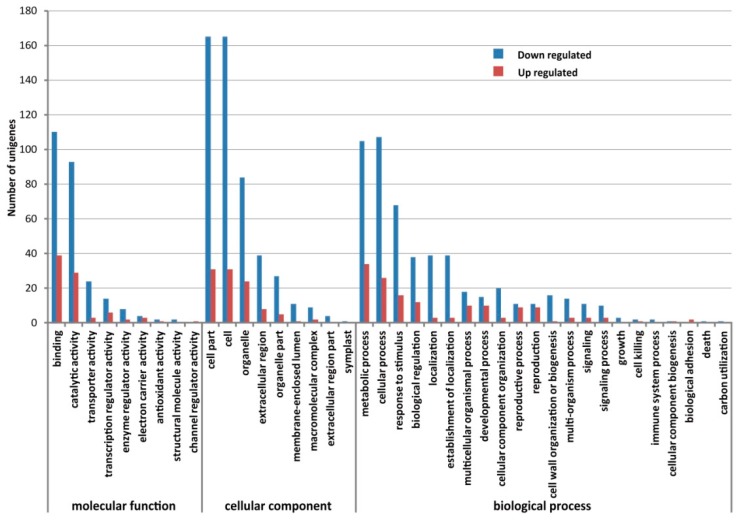
Gene Ontology classification of down- and up-regulated unigenes in floral buds in CMS lines compared with maintainer lines. The *x*-axis indicates the sub-categories; the *y*-axis indicates the number of unigenes in a sub-category.

RNA-Seq gene expression levels were measured using reads per kilobase of exon model per million mapped sequence reads (RPKM) [32]. RPKM values for each gene were compared between the CMS and corresponding maintainer lines to screen for DEGs. We identified 3843 DEGs (2487 up-regulated and 1356 down-regulated genes) in HYBP-A floral buds compared with HYBP-B and 2041 DEGs (676 up-regulated and 1365 down-regulated genes) in YH-A floral buds compared with YH-B. Of these, 539 DEGs exhibited the same direction of expression change in the two CMS lines relative to their corresponding maintainer lines. The transcription level of a total of 127 transcripts was increased and 412 transcripts was decreased in both HYBP-A and YH-A compared with their corresponding maintainer lines (Table S2). These data suggest that combination analysis of two groups of CMS/maintainer lines with different nuclear background was useful to decrease false positives. GO annotation for these DEGs showed that 268 genes were categorized into 50 functional groups with 1629 functional terms indicating that some genes were annotated with multiple terms (Table S3). The DEGs were assigned into nine subcategories in the “molecular function” category, and the two most abundant terms were ‘binding” (149 members) and “catalytic activity” (122 members) (Figure 6). Among “cellular component”, “cell” (199 members), “cell part” (199 members) and “organelle” (108 members) were the three most strongly represented terms (Figure 6). For “biological process”, the three predominant terms for these DEGs were “metabolic process” (141 members), “cellular process” (135 members), “response to stimulus” (83 members) followed by “biological regulation” (50 members) (Figure 6).

There are a total of 79 protein-encoding genes from the radish chloroplast (cp) genome [33], and we retrieved transcripts for 72 cp protein-encoding genes from radish floral buds at this work (transcripts for cp protein genes, *psaJ*, *petG*, *ndhK*, *psaB*, *rps14*, *psbC* and *psbD*, were not detected). In contrast, mitochondrial (mt) genome from radish contains 34 protein genes [34], but only 16 protein transcripts (*atp1*, *atp4*, *atp6*, *atp9*, *ccmFC*, *cob*, *cox2*, *cox3*, *nad4*, *nad4L*, *nad5*, *nad9*, *orf138*, *rpL16*, *rps3* and *rps4*) were detected from the transcriptome. No difference in levels of transcripts between CMS and maintainer lines was found from these annotated mt and cp transcripts in the present work, except CMS-inducing mt *orf138,* which was only expressed in the CMS lines.

Transcription factors (TFs) play important roles in complex biological processes under a wide range of environmental signals by regulating gene transcription through binding to specific DNA sequences in the promoters of multiple target genes [35]. At least 608 transcription factors (TFs) from *Arabidopsis* divided into 34 families were identified in the male gametophyte [36]. Here, 25 TF genes from 11 gene families were down regulated in the radish CMS lines. The tapetum directly surrounding the developing male gametophyte plays a crucial role in the microspore development and maturation by supplying necessary nutrients and structural components. In both rice and *Arabidopsis* at least two bHLH factors (UDT1 and TDR, and DYT1 and AMS, respectively) were shown to be important in tapetum development [37,38,39,40]. The timing of tapetal PCD is crucial for microspore development. T-DNA knockout of *Arabidopsis* *MYB80* (encoding an MYB transcription factor) triggers premature tapetal PCD resulting in male sterility, indicating that *MYB80* is important for tapetal PCD at the proper developmental stage [41]. In the present study, three bHLH and three MYB genes were found to be down-regulated in CMS lines. Since functions of their corresponding *Arabidopsis* genes (AT1G26610, AT3G19580, AT5G04340, AT3G50060, AT5G52660, and AT4G37260) are still unkown, it is worth studying whether these genes are involved in radish CMS.

To date, two C_2_H_2_ zinc-finger transcription factors (DAZ1 and DAZ2) and two WRKY transcription factors (WRKY34 and WRKY2) in *Arabidopsis* are responsible for male gametogenesis. DAZ1 and DAZ2 promote G2- to M-phase transition in pollen [42]. WRKY34 and WRKY2 are required for pollen development at early stage [43]. Since the abortive phenotype for our radish CMS is cytologically observed shortly after the tetrad stage, it remains to be determined whether three DEGs encoding a C_2_H_2_ zinc-finger transcription factor and two WRKYs are involved in early events of the radish CMS.

Heat shock transcription factors (HSFs) and heat shock proteins (HSPs) play key roles in signal transduction in the heat stress response. Several studies suggest that some HSFs and HSPs are related to gametophyte development in the absence of heat stress [44,45,46]. In current work, five HSF and 19 HSP genes were down-regulated in radish CMS lines. Especially, AtHSP23.6 is targeted to mitochondria [47], and the maize homolog *hsp81* is strongly expressed at the pre-meiotic and meiotic stages of microspore development without heat shock [45].

Many fertility restorer genes for the plant CMSs gene were identified as belonging to the PPR family [8,17,18,19,20,21,22]. In this work, three PPR genes were downregulated in the CMS lines. Their orthologs (GenBank accession numbers AT2G20540, AT1G33350 and AT1G71060) from *Arabidopsis* were predicted to target to the mitochondrion. Interestingly, one ortholog *MEF21* from AT2G20540 is involved in the C-to-U transcript editing of the mitochondrial gene *cox3* [48]. These PPR DEGs would be very valuable for further study of their roles in the radish CMS.

### 2.5. Validation of the Expression Difference of DEGs

In order to validate the expression profiling by Illumina sequencing, the expression levels of 10 genes, which showed differential expression between the CMS line HYBP-A and maintainer line HYBP-B, were further analyzed by qRT-PCR. The results showed that the direction of expression change of all the selected genes based on qRT-PCR agreed with those detected by Illumina sequencing (Figure S1). However, the fold changes in expression levels of some genes were different depending on the detection method.

## 3. Experimental Section

### 3.1. Plant Materials and RNA Extraction

The male sterile line 9802A1 possesses the CMS-inducing mitochondrial gene *orf138* [49]. CMS of 9802A1 was introduced in two maintainer lines (HYBP-B and YH-B) by a repeated backcross program (16 generations) using the maintainer lines as recurrent parents. Thus, the resulting two lines, HYBP-A and YH-A, have the same nuclear genomes as HYBP-B and YH-B, respectively. HYBP-B has round red-skinned root and YH-B is an elongated white variety, indicating a different genetic background between the two lines.

### 3.2. Histological Analysis

Fresh floral buds were stored overnight in a formalin-acetic acid-alcohol (FAA) fixative solution. Fixed buds were dehydrated through a series of differently graded ethanol ranging from 50% to 100%. After the length was rapidly measured, dehydrated buds were embedded into Technovit 7100 (Heraeus Kulzer, Wehrheim, Germany) and sectioned with glass knives in a Leica ultracut R ultramicrotome (Leica Microsystems, Wetzlar, Germany). Serial sections (1.5 μm in thickness) of anther tissues were heat fixed to glass slides and stained with 2% toluidine blue. Sectioned anthers were observed with an Olympus BX61 microscope equipped with a colour charge-coupled device (CCD) camera.

### 3.3. RNA Extraction and RNA-Seq

Floral buds about 1.5 mm in length from HYBP-A, HYBP-B, YH-A and YH-B, were sampled, immediately frozen in liquid nitrogen and kept at −80 °C for RNA extraction. Total RNA was isolated using an E.Z.N.A. Plant RNA Kit (OMEGA Bio-Tek, Norcross, GA, USA) according to the manufacturer’s protocol. RNA samples were treated with DNase I at 37 °C for 30 min to remove DNA. A fragmentation buffer was added to break the mRNA-enriched RNAs into short pieces. With these short fragments as templates, first strands of cDNAs were synthesized using random hexamer primers. The second-strand cDNA was generated using the Second Strand Synthesis Reaction Buffer and the Second Strand Synthesis Enzyme Mix from NEBNext^®^ Ultra™ RNA Library Prep Kit for Illumina (NEB, Ipswich, MA, USA). Short fragments were purified with AMPure XP Beads (Agencourt, Beverly, MA, USA) for end repair and tailing A. The resulting A-tailed fragments were mixed with NEBNext Adaptor and Blunt/TA Ligase Master Mix for adaptor ligation. Finally, the sequencing libraries were constructed following PCR amplification with these adaptor-ligated fragments. Paired-end sequencing was then performed using the Illumina sequencing platform (HiSeq™ 2500) according to the manufacturer’s instructions (Illumina, San Diego, CA, USA). Illumina sequencing was performed at Shanghai Hanyu Bio-Tech Co., Ltd., in Shanghai (China).

### 3.4. De Novo Assembly and Database Search

Reads from each library were assembled together. After trimming adapter sequences and filtering low-quality reads, the clean reads were assembled using the software package Trinity consisting of three components: Inchworm, Chrysalis and Butterfly [50]. For radish transcriptome unigene annotation, the assembled unigenes were searched against five public databases using NCBI BLASTX: the Swiss-Prot protein, Gene Ontology (GO), NCBI non-redundant protein sequences (NR), the Kyoto Encyclopedia of Genes and Genomes (KEGG) and eukaryotic ortholog groups (KOG), with an *E*-value threshold of 10^−5^.

### 3.5. Differential Gene Expression

Gene expression levels were measured during the RNA-Seq analysis in RPKM [32]. The DEseq software was used to identify differentially expressed genes in pair-wise comparisons and the results of all statistical tests were corrected for multiple testing using the Benjamini-Hochberg false discovery rate (FDR < 0.001) [51]. Sequences were deemed to be significantly differentially expressed if the adjusted *p*-value obtained by this method (*i.e.*, *Q*-value) was <0.001 and there was at least a two-fold change (>1 or <−1 in log2-ratio value which was calculated dividing the RPKM value of the CMS line by the RPKM value of the maintainer line). The data was deposited at the Gene Expression Omnibus (GEO) database under accession number GSE65142.

GO function enrichment analyses were used to identify markedly enriched function categories, in differentially expressed genes compared with the whole genome background. The formula for the calculation is as follows:

(1) $$p=1-\overset{m-1}{\underset{i=0}{\sum }}\frac{\left(\begin{array}{c} M\\ i \end{array}\right)\left(\begin{array}{c} N-M\\ n-i \end{array}\right)}{\left(\begin{array}{c} N\\ n \end{array}\right)}$$

where, *N* is the number of all genes with GO annotation, *n* is the number of DEGs in *N*, *M* is the number of all genes annotated to specific GO functional category, and *m* is the number of DEGs in *M*.

### 3.6. Real-Time Quantitative PCR (qRT-PCR) Analysis

qRT-PCR was performed using an optical 96-well plate with an iQ5 multicolor real-time PCR system (Bio-RAD, Hercules, CA, USA). Each reaction contained 1.0 μL of cDNA template from the reverse-transcribed reaction mentioned above, 10 nM of gene-specific primers, and 10 μL of iTaq™ Universal SYBR Green supermix (Bio-RAD) in a final volume of 20 μL. The primer sequence of DEGs and the control gene were listed in Table S1. The thermal cycle used was as follows: 95 °C for 30 s, followed by 40 cycles of 95 °C for 15 s, 60 °C for 30 s. qRT-PCR was performed in triplicate for each sample. The comparative threshold (*C*_t_) cycle method was used for determination of relative transcript levels normalized to the actin gene.

## 4. Conclusions

A global view of differential expression of genes from floral buds between radish CMS and corresponding maintainer lines was analyzed in this work. These results provide support for the study of the molecular mechanism of radish CMS.

## Supplementary Materials

Supplementary materials can be found at http://www.mdpi.com/1422-0067/17/1/42/s1.
