# Supplementary material for: Comparative Transcriptome Profile of the Cytoplasmic Male Sterile and Fertile Floral Buds of Radish (Raphanus sativus L.)
Source: Int J Mol Sci. 2016 Jan 6;17(1):42. doi: 10.3390/ijms17010042 (PMC4730287; doi:10.3390/ijms17010042)
Supplement: Supplementary file 1 [file ijms-17-00042-s001.zip › ijms-106164-Supplementary Materials/ijms-106164-Supplementary Materials.pdf]

Supplementary Materials: Comparative Transcriptome Profile of the Cytoplasmic Male Sterile and Fertile Floral Buds of Radish (*Raphanus sativus* L.)

Shiyong Mei, Touming Liu and Zhiwei Wang

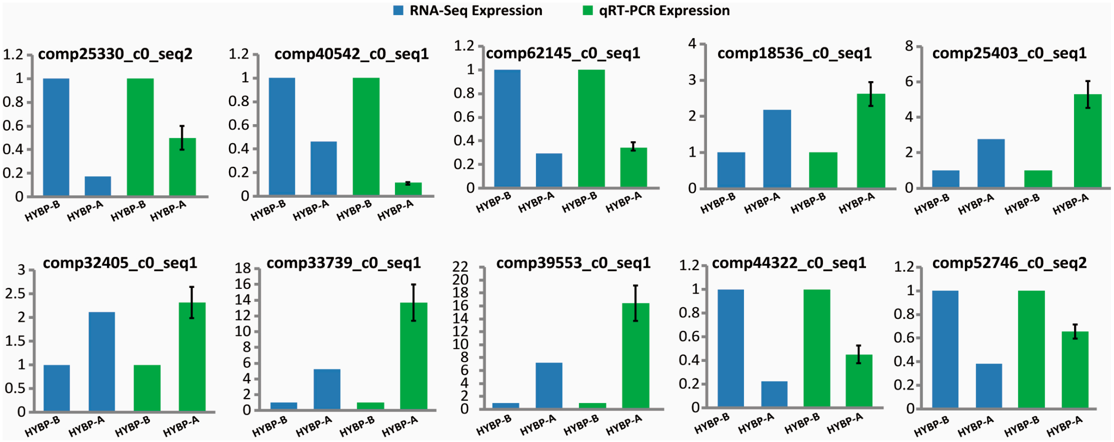

Figure S1. qRT-PCR verification of differentially expressed genes. RNA-Seq expression data come from one sample of HYBP-A and HYBP-B. Transcript levels from qRT-PCR were normalized to the actin gene and given as fold change compared with HYBP-B. Error bars represent SEM (standard error of mean) derived from three technical repeats.

Table S1. Primers for qRT-PCR.

Genes	qRT-PCR Primers
comp25330_c0_seq2	TTCAGACGCAGATTCGTCAC<br>TTCAAGGACATTTGGGTTGG
comp40542_c0_seq1	TCATTATTTCATGCGCCAAC<br>AAGGCAGGAAGACAAGATCG
comp62145_c0_seq1	AATCAATCCCAGCACAATCC<br>TGTTGCAGCATTGGTCAGTT
comp18536_c0_seq1	TGCTGCTTGAGATGTTGGAC<br>CGAAGAAGAACCGGATGAAA
comp25403_c0_seq1	GGGTTCGAGCTTGTTCTCAA<br>TCCACAGATGAGCAGAGTCG
comp32405_c0_seq1	ACCGGTTCGTGACATTAACA<br>ACAAAACTCACCCCAACCAG
comp33739_c0_seq1	ACCCTCATCGACAATCCAAG<br>CCCGTTGAAAATGAATCCAG
comp39553_c0_seq1	CACGACAGTCAGGAAGCTCA<br>AAGTTCCGACAAACGGTCAC
comp44322_c0_seq1	TGGAAACGGAGGGTATGATG<br>ATTCAATCACCACAGCACCA
comp52746_c0_seq2	GATCCTCCTCCGTCGAAGAC<br>TCAAGCGAAAGTTCGTAGCC
